# Is the Mediterranean Diet the Best Approach to NAFLD Treatment Today?

**DOI:** 10.3390/nu13030739

**Published:** 2021-02-26

**Authors:** Francesco Angelico, Domenico Ferro, Francesco Baratta

**Affiliations:** 1 Department of Public Health and Infectious Diseases, Sapienza University of Rome, 00185 Roma, Italy; francesco.baratta@uniroma1.it; 2 Department of Clinical Internal, Anestesiological and Cardiovascular Sciences, Sapienza University of Rome, 00185 Roma, Italy; domenico.ferro@uniroma1.it

Non-alcoholic fatty liver disease (NAFLD) is one of the most prevalent diseases worldwide, involving about 20–30% of the general population. The spread of NAFLD is strongly associated with the increasing prevalence of obesity and type 2 diabetes, especially in Western countries, due to changing lifestyles and dietary habits [1]. NAFLD is the leading cause of cryptogenic cirrhosis, the first cause of liver transplantation in men and the second one in women, and a well-known risk factor for cardiovascular disease, the main cause of mortality and morbidity in this clinical setting [2].

For these reasons, during the last two decades, researchers have conducted intensive research to better understand NAFLD pathophysiology and to discover the best drug for treating NAFLD. However, despite the great efforts, to date there are no therapies currently approved by the Food and Drug Administration (FDA) and European Medicines Agency (EMA) for treating NAFLD.

Today, the only approved therapeutic approach to NAFLD is an intensive therapeutic lifestyle approach, leading to a weight loss of at least 7% [3]. However, the better nutritional approach to obtain weight loss in these patients, together with increasing physical activity, is still debated.

Many of the dietary and nutraceutical approaches proposed for NAFLD are derived from the knowledge we have acquired on the pathogenesis of NAFLD. In fact, all the mechanisms summarized in the “multiple hits” hypothesis [4], such as oxidative stress [5], low grade inflammation [6], dyslipidemia [7], hypovitaminosis E [8], microbiota and dysregulation [9], may well represent a target in the nutritional approach to NAFLD.

In this Special Issue, the Authors analyzed the role of diet in natural history of NAFLD and tried to propose different nutraceutical approaches to specific targets involved in NAFLD pathogenesis. The Special Issue includes original research studies and literature reviews with particular focus to the role of the Mediterranean diet (Med-diet).

Baratta F. et al. [10] showed that poor adherence to the Med-diet and serum lipopolysaccharide (LPS) are associated with oxidative stress in patients with NAFLD. Oxidative stress is one of the key mediators of hepatic damage and according to “the two-hit hypothesis”, is also a major player in the progression from NAFLD to non-alcoholic fatty liver disease (NASH). It has been demonstrated that the increased generation of reactive oxygen species (ROS) can induce lipid peroxidation, leading to inflammation and fibrogenesis through the activation of stellate cells. Lipid peroxidation is increased in NAFLD and can promote inflammation and tissue damage.

In this study, adherence to the Med-diet was investigated by a nine-item validated dietary questionnaire. Serum sNox2-dp and LPS were higher in patients with NAFLD compared to those without it (25.0 vs. 9.0 pg/mL, *p* < 0.001 and 62.0 vs. 44.9 pg/mL, *p* < 0.001, respectively). In patients with NAFLD, the highest sNox2-dp tertile was associated with the top serum LPS tertile (Odds Ratio (OR): 4.71; *p* < 0.001), APRI > 0.7 (OR: 6.96; *p* = 0.005), and Med-diet-score > 6 (OR: 0.14; *p* = 0.026). Analyzing individual foods, the daily consumption of wine (OR: 0.29, *p* = 0.046) and the adequate weekly consumption of fish (OR: 0.32, *p* = 0.030) inversely correlated with the top sNox2-dp tertile. In conclusion, patients with NAFLD showed impaired oxidative stress. Levels of sNox2 correlated with serum LPS and with a low adherence to the Med-Diet.

In order to assess the importance of enhanced oxidant stress, as the link between gut microbiome alterations and increased cardiovascular risk in NAFLD, Ferro D. et al. [11] reported on the role of gut-derived LPS and oxidative stress in the pathogenesis of NAFLD. Oxidative stress, which has previously been described as overexpressed in cardiovascular disease, could represent the link between LPS and the increased cardiovascular risk in NAFLD subjects. In this publication, different therapeutic approaches to reducing oxidative stress and LPS levels in NAFLD are discussed. Many antibiotics have regulatory effects on intestinal microbiota and can reduce serum aspartate aminotransferase (AST), alanine aminotransferase (ALT), and tumor necrosis factor alpha (TNF-α) in NASH animal models. Short-term administration of rifaximin (1200 mg/day for 28 days) improved the clinical status of patients with NAFLD/NASH, which was associated with reduced serum transaminases and circulating endotoxins. Regarding the oxidant status, Med-diet has been reported to reduce oxidant stress, while vitamin E at high daily dosages induced the resolution of NASH in 36% of treated patients. Silymarin had the positive effect of reducing transaminase levels in NAFLD patients and long-term treatment may also decrease fibrosis and slow liver disease progression in NASH. However, the influence of nutraceuticals on gut microbiota and oxidant stress in NAFLD patients has not yet been well elucidated and there are insufficient data either to support or refuse their use in these subjects.

The role of microbiota in NAFLD is of great interest and its modulation has been widely proposed as a nutraceutical approach to NAFLD. Pérez–Montes de Oca A. et al. [12] investigated the possible relationships between microbiota, dietary fiber (DF) and NAFLD. DF supplementation with oligofructose facilitates weight loss, enhances the production of beneficial metabolites, decreases some pathogenic bacteria populations by increasing *Bifidobacteria*, and has effects on intestinal barrier permeability. DF use has been associated with improvement in diverse metabolic diseases, including NAFLD, by modifying gut microbiota. Additionally, it has been shown that a higher insoluble fiber consumption (≥7.5 g/day) revealed improvements in 3 different scores of liver fibrosis. Further research is needed, but based on current evidence, it is reasonable to prescribe DF consumption in early stages of NAFLD to prevent disease progression.

The role of different components of the Med-Diet in NAFLD has not been fully elucidated. A typical food included in the Med-Diet is nuts. Torres M.C.P. et al. [13] asked if nuts are safe in patients with NAFLD. The Med-diet is rich in macro- and micro-nutrients known for their effectiveness in health-promotion and cardio-vascular disease prevention. Moreover, the Med-diet is characterized by the inclusion of nuts. These foods have shown potential benefits in health-promotion as they are rich in DF, which has lipid-lowering effects, and in mono- and poly-unsaturated fatty acids, which help to reduce insulin-resistance and serum cholesterol; besides, nuts contain antioxidants, which may reduce oxidative stress and inflammation. Additionally, nuts are also associated with a better control, or reduction, of body mass index (BMI). All these effects are useful targets to achieve in NAFLD; therefore, nuts have been proposed as a suitable dietary treatment supplement for weight and metabolic control in these patients. In recent years, health authorities raised an alert on nuts consumption, as these may be at high risk of aflatoxin (AF) contamination, for which the controls and legislation are different among countries. AF is a well-known cancerogenic agent and a recognized risk factor for hepatocellular carcinoma. Patients with NAFLD have an overall, inherent sevenfold increased risk of developing hepatocellular carcinoma as compared with the general population. In this context, one could argue that recommending the inclusion of nuts in the diet of NAFLD patients must be balanced with the risk of potential chronic exposure to AF, and every effort should be pursued to assure the safety of these nutrients. In this review, the Authors aimed to summarize the benefits of nuts consumption, the evidence for AF contamination of nuts, and the consequent potential risks in patients with NAFLD.

Med-diet is not only a food pattern, but it also represents a lifestyle pattern [14]. In recent decades, there have been important changes to the Med-diet, such as the reduction of exposure to sunlight, which led to the decrease in serum levels of vitamin D. Barchetta I. et al. [15] showed an update about Vitamin D and metabolic dysfunction-associated fatty liver disease (MAFLD). Recently, it has been proposed to rename NAFLD as MAFLD [16]. This new definition has the advantage of moving from a negative to an inclusive definition which clearly underlines the importance of obesity, diabetes, and metabolic syndrome in the pathogenesis of fatty liver. Vitamin D is a molecule with extensive anti-fibrotic, anti-inflammatory, and insulin-sensitizing properties, which have also been proven to occur in hepatic cells and are involved in immune-metabolic pathways within the gut–adipose tissue–liver axis. Epidemiological data support a relationship between hypovitaminosis D and the presence of NAFLD and NASH; however, results from vitamin D supplementation trials on liver outcomes are controversial [17]. This narrative review provides an overview of the latest evidence on pathophysiological pathways connecting vitamin D to NAFLD, with emphasis on the effects of vitamin D treatment in MAFLD by a nonsystematic literature review of PubMed published clinical trials. Evidence so far available supports the hypothesis of potential benefits of vitamin D supplementation in selected populations of NAFLD patients, as those with shorter disease duration and mild to moderate liver damage.

However, the Med-diet, and its supplementation with nutraceuticals, are not the only dietary approaches proposed to NAFLD. For example, the role of gender differences in response to diets is still under debate. D’Abbondanza M. et al. [18] discussed sex differences in the effects of the very low-carbohydrate ketogenic diet (VLCKD) for the treatment of severe obesity and associated NAFLD. Anthropometric parameters, bioimpedentiometry, degree of liver steatosis measured by ultrasonography, liver function tests, and glucose homeostasis were measured before and after the VLCKD. Males experienced a significantly larger excess body weight loss (EBWL) and a greater reduction in γ-glutamyl transferase (γGT) than females. After dividing the female group by menopausal status, a significant difference between males and pre-menopausal females was found for both EBWL and γGT. No significant difference between groups was observed for improvement in the Edmonton stage or in the degree of steatosis. They concluded that the efficacy of following a VLCKD in severe obesity is affected by sex differences and, for females, by menopausal status. Males seem to experience larger benefits than females in terms of EBWL and NAFLD improvement. These differences are attenuated after menopause, probably because of changes in hormonal profile and body composition.

Conversely, diets rich in protein, such as the proposed VLCKD, could be a critical protein source in patients with cirrhosis, the final stage of NAFLD. Iqbal U. et al. [19] proposed a comprehensive review evaluating the impact of protein source (vegetarian vs. meat-based) in hepatic encephalopathy (HE). Patients with cirrhosis are at a high risk for protein-calorie malnutrition due to altered metabolism. Current evidence has changed the old belief of protein restriction in patients with cirrhosis and now 1.2 to 1.5 g/kg/day protein intake is recommended. Case series and studies with small numbers of participants showed that a vegetarian protein diet decreases the symptoms of HE when compared to a meat-based diet, but the evidence is limited and requires further larger randomized controlled trials. However, vegetable or milk-based protein diets are good substitutes for patients averse to meat intake. Branch chain amino acids (BCAA) (leucine, isoleucine, and valine) have also been shown to be effective in alleviating symptoms of HE and are recommended as an alternative therapy in patients with cirrhosis for the treatment of HE. In this review, they provided an overview of current literature evaluating the role of protein intake in the management of HE in cirrhosis.

In addition to the Med-Diet, another dietary supplementation against microbiota dysregulation and consequent NAFLD was proposed by Nakano H. et al. [20] in this Special Issue. These Authors reported that Bilberry Anthocyanins (BA) ameliorate NAFLD by improving dyslipidemia and gut microbiome dysbiosis. The results revealed that supplementation with BA attenuated the serum levels of AST, ALT, low-density lipoprotein, cholesterol (LDL-c), fat content in liver, 2-thiobarbituric acid reactive substances (TBARS) and α-smooth muscle actin (α-SMA) caused by the western diet. Furthermore, gut microbiota characterized by 16S rRNA sequencing revealed that BA reduced remarkably the ratio of Firmicutes/Bacteroidetes (F/B) and modified gut microbiome. In particular, BA increased the relative abundance of g_*Akkermansia* and g_*Parabacteroides*. Taken together, these data demonstrated that BA might ameliorate WD-induced NAFLD by attenuating dyslipidemia and gut microbiome dysbiosis.

In addition, Oppedisano F. et al. [21] discussed the protective effect of Cynara Cardunculus extract (CyC) in diet-induced NAFLD and the involvement of OCTN1 and OCTN2 Transporter Subfamily. In the study they used a wild type of CyC, rich in sesquiterpens and antioxidant active ingredients, in rats fed a high fat diet (HFD) compared to a normal fat diet (NFD). In rats fed HFD for four consecutive weeks, they found a significant increase of serum cholesterol, triglyceride, and glucose. This effect was accompanied by increased body weight and by histopathological features of liver steatosis. The alterations of metabolic parameters found in HFDs were antagonized dose-dependently by daily oral supplementation of rats with CyC 10 and 20 mg/kg over four weeks, an effect associated to significant improvement of liver steatosis. The effect of CyC (20 mg/kg) was also associated to enhanced expression of both OCTN1 and OCTN2 carnitine-linked transporters. Thus, these data suggested a contribution of carnitine system in the protective effect of CyC in diet-induced hyperlipidemia, insulin-resistance, and NAFLD.

As demonstrated by the numerous contributions submitted to this Special Issue, in the recent years, the beneficial effects of nutraceuticals on NAFLD progression have received increasing attention and several types of nutraceuticals have been suggested for the treatment of NAFLD/NASH. For some of them, several clinical trials highlighted an improvement of serum liver enzymes and a possible positive influence on liver histology. Pani A. et al. [22], to resume the published data, proposed a systematic review on deficiencies and supplementation of inositol (INS) in NAFLD. They performed a systematic review of the literature to find preclinical and clinical evidence of INS supplementation efficacy in NAFLD patients. They retrieved 10 studies on animal models assessing Myoinosiol or Pinitol deficiency or supplementation and one human randomized controlled trial (RCT). Overall, INS deficiency was associated with increased fatty liver in animals. Conversely, INS supplementation in animal models of fatty liver reduced hepatic triglycerides and cholesterol accumulation and maintained a normal ultrastructural liver histopathology. In the one model that included RCT, Pinitol supplementation obtained similar results. Pinitol significantly reduced liver fat, post-prandial triglycerides, AST levels, and lipid peroxidation increasing glutathione peroxidase activity. These results, despite being limited, indicate the need for further evaluation of INS in NAFLD in larger clinical trials.

Finally, beyond the dietary intervention, the lifestyle therapeutic approach to NAFLD may have a benefit from to an improvement of life quality. Han A. L. [22] reported about the association between NAFLD, dietary habits, stress, and health-related quality of life in Korean adults (HRQoL). They performed a complex sample logistic regression analysis and estimated the odds ratios by adjusting for significant factors to evaluate associations between NAFLD and dietary habits, stress, and HRQoL. Occurrence of NAFLD was not significantly associated with meal frequencies over one week. With an increase in stress, based on the stress perception rate, the risk of NAFLD increased 1.3-fold (95% confidence interval (CI): 1.1–1.4, *p* < 0.05). Thus, NAFLD treatment should include stress management, and underlying HRQoL should be considered during treatment.

In conclusion, for an effective drug against NAFLD to be available, dietary, nutraceutical, and lifestyle approaches are the most important tools for patients with NAFLD. In our opinion, while waiting for a magic bullet against NAFLD, the Med-Diet, as also suggested by the European guidelines [3], is the Columbus egg for the nutritional approach to patients with NAFLD.
